# Application of machine learning to structural connectome to predict symptom reduction in depressed adolescents with cognitive behavioral therapy (CBT)

**DOI:** 10.1016/j.nicl.2019.101914

**Published:** 2019-07-02

**Authors:** Olga Tymofiyeva, Justin P. Yuan, Chiung-Yu Huang, Colm G. Connolly, Eva Henje Blom, Duan Xu, Tony T. Yang

**Affiliations:** a Department of Radiology & Biomedical Imaging, University of California, San Francisco, 1700 4th Street, BH102, San Francisco, CA 94143, USA; b Department of Epidemiology and Biostatistics, University of California, San Francisco, 550 16th Street, San Francisco, CA 94143, USA; c Department of Psychiatry and the Langley Porter Psychiatric Institute, Division of Child and Adolescent Psychiatry, Weill Institute for Neurosciences, University of California, San Francisco, 401 Parnassus Avenue, San Francisco, CA 94143, USA; d Department of Biomedical Sciences, Florida State University College of Medicine, 1115 West Call Street, Tallahassee, FL 32306, USA; e Department of Clinical Science/Child- and Adolescent Psychiatry, Umeå University, SE-901 87 Umeå, Sweden

**Keywords:** Machine learning, Adolescent depression, Diffusion MRI, Connectomics, Brain network, CBT

## Abstract

**Purpose:**

Adolescent major depressive disorder (MDD) is a highly prevalent, incapacitating and costly illness. Many depressed teens do not improve with cognitive behavioral therapy (CBT), a first-line treatment for adolescent MDD, and face devastating consequences of increased risk of suicide and many negative health outcomes. “Who will improve with CBT?” is a crucial question that remains unanswered, and treatment planning for adolescent depression remains biologically unguided. The purpose of this study was to utilize machine learning applied to patients' brain imaging data in order to help predict depressive symptom reduction with CBT.

**Methods:**

We applied supervised machine learning to diffusion MRI-based structural connectome data in order to predict symptom reduction in 30 depressed adolescents after three months of CBT. A set of 21 attributes was chosen, including the baseline depression score, age, gender, two global network properties, and node strengths of brain regions previously implicated in depression. The practical and robust J48 pruned tree classifier was utilized with a 10-fold cross-validation.

**Results:**

The classification resulted in an 83% accuracy of predicting depressive symptom reduction. The resulting tree of size seven with only three attributes highlights the role of the right thalamus in predicting depressive symptom reduction with CBT. Additional analysis showed a significant negative correlation between the change in the depressive symptoms and the node strength of the right thalamus.

**Conclusions:**

Our results demonstrate that a machine learning algorithm that exclusively uses structural connectome data and the baseline depression score can predict with a high accuracy depressive symptom reduction in adolescent MDD with CBT. This knowledge can help improve treatment planning for adolescent depression.

## Highlights

• Machine learning predicted symptom reduction in depressed teens with 83% accuracy.
• Resulting prunned classification tree size was 7, with only 3 attributes.
• Change in depression symptoms correlated with node strength of the right thalamus.

## 1 Introduction

Adolescent major depressive disorder (MDD) is increasingly recognized as a highly prevalent, incapacitating, and costly illness. The lifetime and 12-month prevalence of adolescent MDD is estimated to be 11% and 7.5%, respectively (Avenevoli et al., 2015). Adolescent depression episodes are often chronic and recurrent, persisting into adulthood (Naicker et al., 2013; Rohde, 2005). The disease has serious negative psychosocial consequences, including but not limited to: impaired academic and work functioning, social difficulties, substance abuse, and suicide (Birmaher and Brent, 2007).

Cognitive behavioral therapy (CBT) is the most widely-researched non-pharmacological approach (Callahan et al., 2012) and one of the most empirically-supported treatments for adolescent MDD (Spirito et al., 2011). The CBT treatment approach is rooted both in behavioral, and cognitive formulations of depression (Ferster, 1966; Beck, 1976; Seligman, 1975; Hetrick et al., 2016). The main goal of the cognitive component is to help the depressed patient become aware of pessimistic, negative, and disproportionately self-blaming thoughts and eventually replace them with more constructive cognitions (Rohde, 2005). The main goal of the behavioral component is to increase engagement in behaviors that either elicit positive reinforcement or avoid negative reinforcement from the environment (Rohde, 2005).

Unfortunately, a large percentage of depressed adolescents do not respond to CBT treatment. CBT has been shown to be effective only for approximately 43% to 65% of adolescent patients suffering from depression (March et al., 2004, 2007). “Who will respond to CBT?” is a crucial question to investigate and our ability to answer it will significantly improve treatment planning and efficacy, and therefore decrease burden for the patients and their support network.

As with treatment-resistant depression in general (when an MDD patient does not respond to multiple or any available standard treatments), responsiveness or non-responsiveness to CBT can be linked to multiple core processes involved in MDD such as stress, genetics and epigenetics, and brain structural and functional plasticity (Akil et al., 2018). Brain imaging, in particular MRI, offers a means to identify potential predictive biomarkers that are grounded in the neurobiology of the treatment and the pathophysiology of adolescent MDD. Several recent studies used MRI in combination with traditional statistical approaches to predict clinical improvement after treatment in adult depression. For example, anterior cingulate volume predicted improvement after CBT in 10 adults with MDD: the degree of improvement in depressive symptoms was positively correlated with gray matter (GM) volume in the caudal portion of the anterior cingulate cortex (Fujino et al., 2015). Other recent studies used functional activation and connectivity during rest or task to predict clinical improvement after treatment. Dunlop et al., analyzed resting-state functional connectivity data using a bilateral subcallosal cingulate cortex (SCC) seed in 122 depressed patients who completed 12 weeks of randomized treatment with CBT or antidepressant medication (Dunlop et al., 2017). The authors achieved overall classification rates of 72%–78% for clinical remission and 75%–89% for treatment failure. Positive summed SCC functional connectivity was associated with clinical remission with CBT and treatment failure with medication, whereas negative summed functional connectivity scores were associated with clinical remission to medication and treatment failure with CBT. In older, depressed adults, functional MRI (fMRI) activation during executive function also predicted clinical improvement after CBT (Thompson et al., 2015).

Along with traditional statistical approaches that suit hypothesis-driven studies, supervised machine learning methods have been gaining popularity as they allow for a data-driven search for brain regions that are most predictive of improvement after clinical treatment. To predict clinical response after treatment in depressed adults, Costafreda et al., used support vector machines (SVMs): a supervised pattern recognition method allowing predictions at the individual level (Costafreda et al., 2009). Patients received antidepressants (18 patients) or CBT (12 patients). The whole brain structural neuroanatomy predicted 89% of the clinical response. Supervised machine learning methods have also been used more recently to predict long-term clinical improvement after a 13-week Internet-delivered CBT (iCBT) in 26 adult patients with social anxiety disorder (SAD) (Månsson et al., 2015), which is highly comorbid with MDD (Angold and Costello, 1993). The authors also used SVMs and trained them to separate long-term responders after treatment from those who failed to respond based on blood oxygen level-dependent (BOLD) responses to self-referential criticism. From multivariate BOLD responses in the dorsal anterior cingulate cortex (dACC) together with the amygdala, they were able to predict long-term clinical response rate after iCBT with an accuracy of 92%.

The analyses in the studies mentioned above were focused on specific brain regions, functional activation or functional connectivity. Apart from the knowledge about involvement of specific regions, the importance of anatomical white matter connections between these regions and their role within the brain network as a whole are becoming increasingly recognized and studied within the framework of MRI connectomics. MRI connectomics treats the brain as a complex network, which can be characterized in terms of local and global properties using graph theory (Hagmann et al., 2010a). MRI connectomics has been applied to both the adult and developing brain (Hagmann et al., 2010b; Tymofiyeva et al., 2013). This framework has also been applied to study the neural signature of adult depression (Sacchet et al., 2016; Qin et al., 2014; Korgaonkar et al., 2014; see Gong and He, 2015 for review), as well as adolescent depression (Tymofiyeva et al., 2017; Ellis et al., 2017) and anxiety (Sharp and Telzer, 2017).

Whitfield-Gabrieli et al., used both, resting-state functional and diffusion MRI-based structural connectivity to predict how well CBT treatment improved anxiety symptoms for SAD in adults (Whitfield-Gabrieli et al., 2016). They found that both brain structure and neural connectivity among different regions predicted how well CBT reduced clinical symptoms. Importantly, clinician estimates of clinical improvement after treatment using a behavioral assessment tool accounted only for 12% of the variance in clinical benefit, but adding information from neuroimaging increased by fivefold the estimates of successful clinical improvement in patients after CBT. It should be noted that in the study by Whitfield-Gabrieli and colleagues diffusion MRI was used only to examine one single track, the right inferior longitudinal fasciculus (ILF), because it was the tract most associated with fMRI-derived occipital-temporal regions predictive of clinical improvement after CBT in SAD patients. Connections among other regions implicated in the disorder may carry additional important information for predicting clinical improvement after CBT. With respect to MDD populations, commonly implicated brain areas in the published literature include cortical regions – the prefrontal cortex (PFC) (e.g., Menon, 2011; Kimbrell et al., 2002; Tymofiyeva et al., 2017), the anterior cingulate cortex (ACC) (e.g., Connolly et al., 2013; Yang et al., 2009; Lichenstein et al., 2016; Ho et al., 2017), the orbital frontal cortex (OFC) (e.g., Cheng et al., 2016), and the insula (e.g., Henje Blom et al., 2015); subcortical limbic brain regions – the amygdala (e.g., Yang et al., 2010; Connolly et al., 2017; Perlman et al., 2012), hippocampus (e.g., Frodl et al., 2006), and the thalamus (e.g., Greicius et al., 2007); and the basal ganglia – the striatum and specifically the caudate (e.g., Kim et al., 2008; Pizzagalli et al., 2009; Tymofiyeva et al., 2017). Connections among these brain regions, as well as connections between these regions and the rest of the brain, can be expected to play a role in predicting clinical improvement after CBT in depressed populations.

To our knowledge, no published studies have attempted to apply a structural MRI connectomics or machine learning approach to predict depressive symptom reduction after CBT in adolescents with MDD. The goal of this study was to assess the accuracy that can be achieved by applying a practical and robust system for decision tree induction called C4.5 (Quinlan, 1993; Frank et al., 2016) to MRI connectomics attributes to predict depressive symptom reduction after CBT in adolescents with MDD. We hypothesized that a machine learning algorithm applied to brain network features will be able to predict improvement with CBT significantly better than the “best guess” based on the class frequency.

## 2 Methods

### 2.1 Participants and clinical information

The study was approved by the Institutional Review Boards at the University of California San Diego (UCSD), University of California San Francisco (UCSF), Rady Children's Hospital in San Diego, and the County of San Diego. All participants provided written informed assent and their parent(s) or legal guardian(s) provided written informed consent in accordance with the Declaration of Helsinki.

The study protocol, recruitment procedures, inclusion/exclusion criteria, clinical assessments, and MRI data acquisition and post-processing have been described previously (LeWinn et al., 2014; Tymofiyeva et al., 2017, Tymofiyeva et al., 2018) and are included here in brief. A subset of 30 postpubertal (Tanner stage 3–5) adolescents with MDD according to the DSM-IV from Tymofiyeva et al. (2017), who received CBT treatment and had a three-month follow-up assessment were included in this study. Among these subjects 15 were female and 15 were male. The mean age at the baseline was 16.0 yrs. (standard deviation 1.3 yrs.; range 13.2–17.8 yrs.). Depressive symptoms were assessed for each participant using the clinician-administered Children's Depression Rating Scale-Revised (CDRS-R) (Poznanski, 1996). This assessment was performed twice: at baseline and after three months, during which the patients underwent CBT treatment. Six of the subjects were receiving antidepressant medication in addition to CBT and 24 subjects were unmedicated.

### 2.2 MRI data acquisition and connectome construction

MRI data were collected at the baseline using a 3 T MRI system (MR750, GE Healthcare, Milwaukee, Wisconsin, USA) at the UCSD Center for Functional Magnetic Resonance Imaging (CFMRI). High-resolution anatomical T1-weighted images were acquired using a fast spoiled gradient recalled (SPGR) pulse sequence (TR/TE = 8.1/3.17 ms, flip angle = 12°, slice thickness = 1 mm, FOV = 250 × 250 mm, matrix = 256 × 256, voxel size = 0.98 × 0.98 × 1 mm). The diffusion-weighted images were acquired using a dual spin echo, single-shot echo-planar imaging (EPI) sequence, 30 directions, b-value = 1500s/mm^2^, TR/TE = 7200/86.5 ms, FOV = 180 × 180 mm, matrix = 96 × 96, voxel size = 1.875 × 1.875 × 2.5 mm, two averages.

**Fig. 1:**
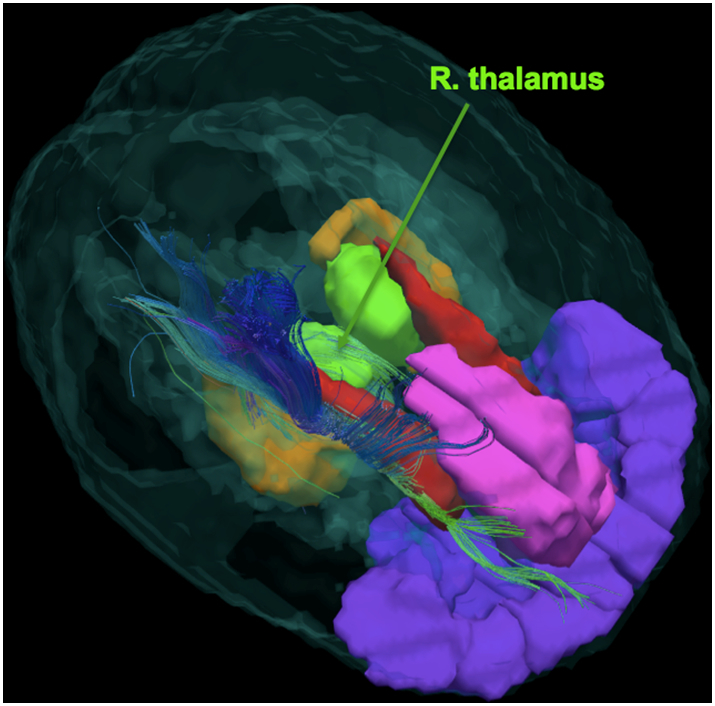
DTI-based tractogram in an adolescent with major depressive disorder (MDD). The image shows streamlines going through the right thalamus (depicted in green). Other structures shown: anterior cingulate cortex (ACC) in pink, orbital frontal cortex (OFC) in purple, caudate in red, and hippocampus in orange.

The T1-weighted images were bias-field-corrected, skull-stripped, and transformed to MNI152 space using an affine transform in FSL (Smith et al., 2004). A DTI quality assurance step and head motion assessment were performed as previously described by our group (LeWinn et al., 2014). DTI reconstruction and deterministic whole-brain streamline fiber tractography were performed using the Diffusion Toolkit (Wang et al., 2007) with Fiber Assignment by Continuous Tracking (FACT) and an angle threshold of 35° (Fig. 1).

**Fig. 2:**
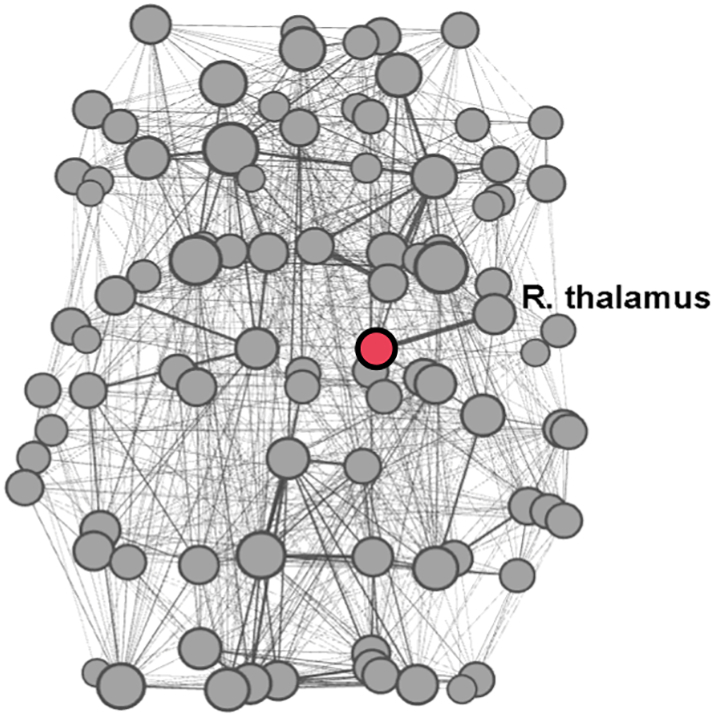
A brain network (connectome) example in an adolescent study participant represented as a graph (set of nodes and edges). The right thalamus node highlighted in red. Size of the nodes is proportional to the node degree. Network visualization was performed using Gephi (Bastian et al., 2009).

Our aim was to construct for each subject the large-scale brain network (connectome) of white matter fiber connections between gray matter structures of the brain and to analyze it as an abstract representation: a graph (set of nodes and edges). Segmentation of the cerebrum into 90 regions of interest (ROIs) was performed in the DTI space using the Automated Anatomical Labeling (AAL) atlas (Tzourio-Mazoyer et al., 2002) and intermediate registration to T1-weighted images in MNI space. The AAL-based ROIs were dilated by one voxel and served as network nodes. Connections between AAL ROIs were calculated by using as weights the average fractional anisotropy (FA) sampled along the connecting streamlines. The FA-weighted connections were recorded as a 90 × 90 connectivity matrix, in which each row/column corresponds to a distinct node (brain ROI) (Fig. 2). The choice of the connectome construction pipeline was based on the previously demonstrated high test-retest reliability of FA-weighted networks in adolescents (Yuan et al., 2018). Local network properties (node strengths) and two most commonly used global network properties (average clustering coefficient and characteristic path length) were assessed using the Brain Connectivity Toolbox (Rubinov and Sporns, 2010). Network visualization was performed using Gephi, an open-source network visualization software package (Bastian et al., 2009).

### 2.3 Machine learning/statistical analyses

To perform a supervised machine learning classification analysis, a set of 21 attributes was selected, including the baseline depressive score, age, gender, two FA-weighted global network properties (average clustering coefficient and characteristic path length), and FA-weighted node strengths of 16 nodes corresponding to eight brain regions (right and left) previously implicated in depression (see introduction): OFC, ACC, middle frontal gyrus (MFG), insula, amygdala, hippocampus, thalamus, and caudate. We aimed to correctly classify patients as belonging to one of the two classes: class 1 – those patients whose depressive symptoms improved after CBT (negative value of (CDRS-R_post-CBT_ - CDRS-R_pre-CBT_)) and class 0 – patients whose depressive symptoms stayed the same or worsened (non-negative value of (CDRS-R_post-CBT_ - CDRS-R_pre-CBT_)). Thus, any positive change in depressive symptoms was interpreted as an improvement, and such a change does not necessarily imply a clinically significant change.

Machine learning analysis was performed using WEKA software (version 3.8.1) developed at the University of Waikato in New Zealand (Frank et al., 2016). The J48 pruned tree classifier (JAVA implementation of the C4.5 algorithm (Quinlan, 1993) in WEKA), which is based on the concept of information entropy, was applied with a 10-fold cross-validation. The experiment was repeated 10,000 times with a new random number generator seed for the split of the dataset into a training and test set chosen independently at each run, using the Weka Experiment Environment. To assess the performance of the J48 classifier, the result was compared with 10,000 runs of the ZeroR algorithm, by using a paired t-test comparison of the obtained accuracies. ZeroR is the simplest classification method, which ignores all attributes and always predicts the majority class (makes the “best guess” based on the class frequency) (Frank et al., 2016). The ZeroR classifier thus provides the baseline accuracy as a benchmark for other classification methods.

Bivariate correlations were calculated using Pearson's correlation coefficient in IBM SPSS Statistics software (version 25).

## 3 Results

Demographic and clinical characteristics of the study participants are summarized in Table 1. Nineteen adolescent patients with MDD showed an improvement of the depressive symptoms (negative value of (CDRS-R_post-CBT_ - CDRS-R_pre-CBT_)), whereas 11 did not show improvement. No between-group differences in the average amount of head motion during the DTI scan were observed between improvers and non-improvers (with independent samples t-tests resulting in p = .12 for rotation and p = .51 for translation). The J48 classification resulted in an 83% average accuracy of predicting improvement of depressive symptoms (standard deviation 23%). The average false positive rate was 15% (standard deviation 27%), the average false negative rate was 20% (standard deviation 39%), the average true positive rate was 80% (standard deviation 39%), and the average true negative rate was 85% (standard deviation 27%). The sensitivity for improvement was 82%, the specificity for improvement was 84%. This result was statistically significantly better than the result of the ZeroR algorithm (p < .0001, assessed using a paired t-test, with 10,000 10-fold cross-validated runs for each of the two algorithms performed in the Weka Experiment Environment).

**Table 1:** Demographic and clinical characteristics of the study participants.

Number of patients	30
Age, yrs. (standard deviation; range)	16.0 (1.3; 13.2–17.8)
Sex (Male/Female)	15/15
Baseline Children's Depression Rating Scale-Revised (CDRS-R) t-score (standard deviation; range)	71.3 (8.5; 55–85)
Percent change in depressive symptoms after CBT treatment, calculated as ∆CDRS-R = (CDRS-R_post-CBT_ - CDRS-R_pre-CBT_)/CDRS-R_pre-CBT_*100% (standard deviation; range)	−6.0 (13.4; −28-18)
CDRS-R t-score improvement after CBT (Yes/No)	19/11

**Fig. 3:**
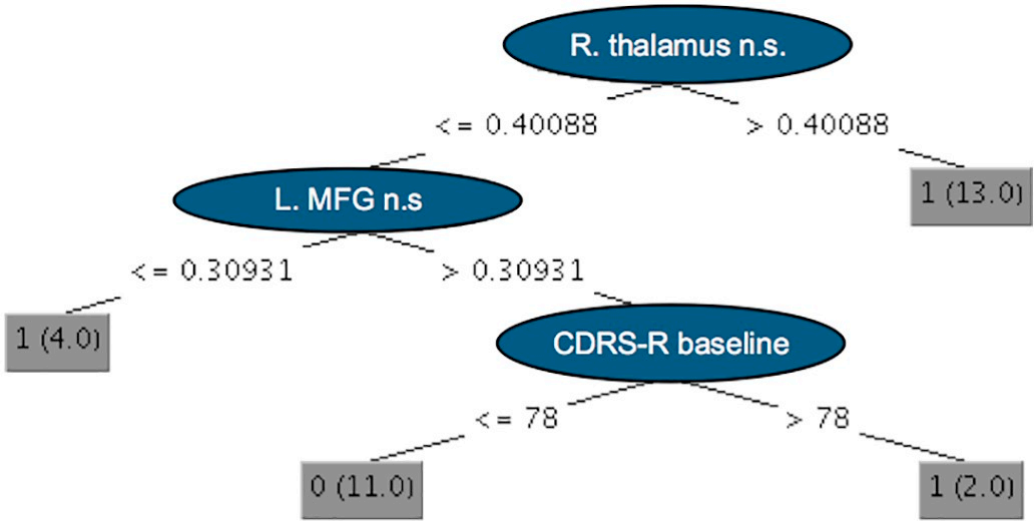
Resulting classification tree with only three attributes (internal nodes) and four terminal nodes called “leaves” (result of the J48 pruned tree classifier implemented in WEKA). The size of the tree is seven, which is calculated by adding together the number of internal nodes and leaves, helping to reveal the complexity of the tree. Achieved accuracy of predicting clinical symptom improvement is 83%. MFG – middle frontal gyrus, n.s. – node strength, R. – right, L. – left, CDRS-R - the Children's Depression Rating Scale-Revised. Class labels: class 1 – patients whose depressive symptoms improved, class 0 – patients whose depressive symptoms stayed the same or worsened.

The resulting tree of size seven with only three attributes (four leaves) (Fig. 3) highlights the role of the right thalamus in predicting depressive symptom reduction after CBT in teens diagnosed with MDD. The other two attributes were the baseline depression score and the node strength of the left MFG.

**Fig. 4:**
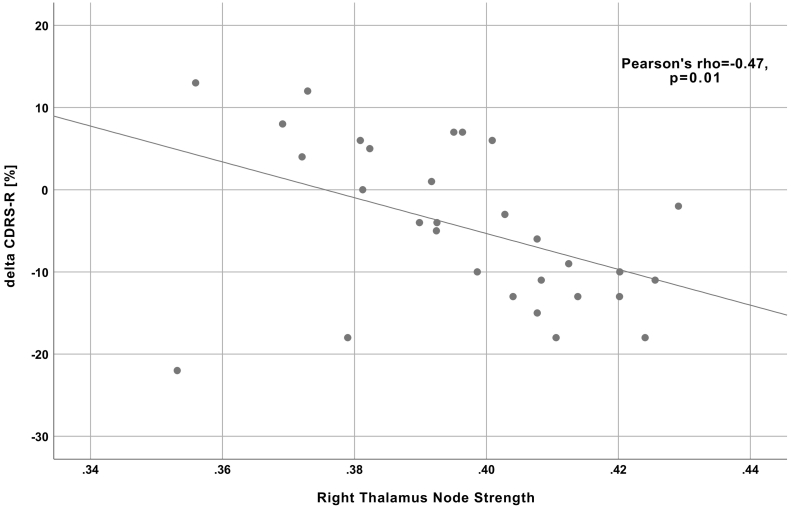
A scatterplot demonstrating correlation between the baseline node strength of the right thalamus and the percent change in depressive symptoms (measured using the Children's Depression Rating Scale-Revised) after CBT.

Additional analysis showed a significant negative correlation between the percent change in the depressive symptoms and the node strength of the right thalamus (Pearson's r of −0.47 (p = .01); Fig. 4). Thus, depressed adolescents with higher node strength of the right thalamus demonstrated better improvement (a more negative percent change in depressive symptoms after CBT treatment, calculated as ∆CDRS-R = (CDRS-R_post-CBT_ - CDRS-R_pre-CBT_)/CDRS-R_pre-CBT_*100%).

When six adolescent patients who were receiving medication in addition to CBT were removed, the J48 algorithm resulted in an identical classification tree, albeit with a lower classification accuracy (54%). The correlation between the percent change in the depressive symptoms and the node strength of the right thalamus remained significant: a Pearson's r of −0.47 (p = .02). Out of the six patients taking medication, five were improvers and one was a non-improver.

## 4 Discussion

Our results demonstrate that a machine learning algorithm that exclusively uses structural connectome data (white matter connectivity) and the baseline depressive score can predict with a high accuracy depressive symptom reduction in MDD adolescents after CBT. This accuracy was achieved by applying the J48 pruned tree classifier (Quinlan, 1993; Frank et al., 2016). It is considered the “work-horse” of machine learning tree algorithms, practical and robust under a wide variety of circumstances (Frank et al., 2016). Compared to the conventional logistic regression modeling, the nonparametric nature of tree-based methods automatically takes nonlinearity and interactions among attributes into consideration.

While interpretability is often a problem in machine learning research (O'Donnell and Schultz, 2015), the resulting classification tree and subsequent correlational analysis in our study offer a possibility to gain insight into the underlying biological mechanisms. Specifically, our results suggest that the microstructural properties of white matter fibers connecting the right thalamus to other brain regions may be compromised in adolescents who do not show any improvement with CBT. As discussed in the introduction, one of the main goals of CBT (the behavioral component) is to increase engagement in behaviors that result in positive reinforcement. However, anhedonic symptoms that are a core clinical characteristic of MDD may be blocking such engagement. Anhedonia, the lack of interest or pleasure in response to hedonic stimuli or experiences, and especially anticipatory anhedonia as opposed to consummatory anhedonia, is a cardinal symptom of depression (Sherdell et al., 2012). Behavior activation (BA) is therefore used to more directly target anhedonic symptoms in part by increasing exposure to and reinforcement of pleasant, rewarding events. However, BA is only used on average 1.5 times during the course of CBT treatment of adolescent depression (Kennard et al., 2009), and the initial ability to generate goal-directed behavior may be pivotal in the effectiveness of CBT. Published research supports that the thalamus is directly involved in anticipatory anhedonia (Li et al., 2015; Knutson et al., 2000; Komura et al., 2001) and in the generation of goal-directed behavior (Haber and Calzavara, 2009), the lack of which can make subsequent CBT treatment ineffective (Alvares et al., 2014).

In our study, the node strength of the left thalamus was not selected by the machine learning algorithm as a predictive attribute. The laterality of our results, specifically, the engagement of the right thalamus, may be explained by the right-hemisphere hypothesis, which posits that the right hemisphere regions are recruited more than their left counterparts in affective processing, regardless of affective valence (Borod et al., 1998).

We only focused on interpreting and further analyzing the first attribute of the J48 classification tree, also known as the “root node” (in our case, the node strength of the right thalamus), due to the limited number of classified instances and reduced interpretability of the other two attributes.

Though one of the first studies to explore the utility of machine learning using a structural connectome to predict response to CBT in adolescents, this study's findings need to be interpreted in the light of its limitations. First, the sample size in our study was limited to 30 adolescents diagnosed with MDD. Future prospective validation studies with a larger number of patients would provide an important test to the cutting-edge methodology used in this study. Second, heterogeneity in CBT treatment protocols (delivery by different providers, with different schedules, etc.) may limit our results. This, however, should also be seen as a potentionally clinically more meaningful and effective treatment approach, as a highly scripted, manualized and standardized CBT delivery format that impedes patient-oriented adaptations by the therapist to the specific needs of individual patients may limit the clinical effectiveness of CBT treatment (March et al., 2004). Nevertheless, a relatively small reduction in depressive symptoms was observed in our study (on average, 6%) (Table 1). Third, we used a binary classification into two classes as the main goal of the study, which inevitably raises the question of how to define the classes: improvers vs. non-improvers. On one hand, small incremental improvement in depressive symptoms might not always be of significant clinical importance. On the other hand, clinical thresholds such as a decrease of at least 50% on the CDRS-R scale as used in clinical trials of teen MDD (Brent et al., 2008) or no longer fulfilling the diagnostic criteria for MDD, might not be neurobiologically meaningful, as they are not based on neuroscience-derived criteria. Future studies might consider transitioning from classification to regression analysis in order to avoid the binarization problem and to provide clinicians with richer information about the percentage change of clinical depression symptoms following CBT. Such information would be useful for the clinician because she or he must often take into account several factors (e.g., severity of depression, patient preferences, accessibility to treatment, etc.) in deciding what is the best clinical treatment for an individual patient. For example, a clinician may be more inclined to select CBT for a mildly depressed teen even if the patient will only improve 30% as compared to a severely depressed teen for whom the clinician will most likely want to see a >50% improvement in depression symptoms after CBT due to the severity of the depression. Our additional analysis of the correlation between the percent change in the depressive symptoms and the node strength of the right thalamus provides this type of useful information (Fig. 4). Finally, since our study design did not include a no-treatment control group, we could not differentiate between treatment-specific and spontaneous symptom reduction. Moreover, without an active comparison treatment group we could not conclude whether the identified imaging biomarkers predict improvements specifically for CBT or across all potential treatments (i.e., a nonspecific predictor). Nevertheless, our results may bring us one step closer to precision psychiatry. While the ultimate goal of precision medicine is to find the optimal treatment for a specific patient, an important milestone in the precision medicine endeavor is to find the optimal patient for a specific treatment (National Institute of Mental Health (NIMH) Priority 3.2.B.6; NIMH, 2018).

In conclusion, our results show that a machine learning algorithm that uses structural connectome data and the baseline depression scores can predict, with a high accuracy, depressive symptom reduction in adolescents with MDD, who are receiving CBT treatment. This knowledge can improve treatment planning for adolescent depression. Equally important, the results shed light on the candidate neurobiological mechanisms underlying the responsiveness to CBT and can help optimize and develop new preventive and therapeutic interventions for adolescent MDD.

## Funding

This study was supported by NIMH R01MH085734 to TTY; by NCCIH R21AT009173 and R61AT009864 to OT, TTY and EHB; by NICHD R01HD072074 to DX and OT; by UCSF Research Evaluation and Allocation Committee (REAC) and J. Jacobson Fund to OT, EHB, TTY and DX; by the Fahs-Beck Fund for Research and Experimentation at The New York Community Trust to OT; by the Swedish Research Council 350-2012-303 to EHB; by the Brain and Behavior Research Foundation (formerly NARSAD) to TTY. The content is solely the responsibility of the authors and does not necessarily represent the official views of the National Institutes of Health of other funding agencies. The funding agencies did not play any role in study design; in the collection, analysis and interpretation of data; in the writing of the report; and in the decision to submit the article for publication.

## Declaration of Competing Interest

The authors have no conflict of interest to disclose.
